# Correction: Autologous P63+ lung progenitor cell transplantation in idiopathic pulmonary fibrosis: a phase 1 clinical trial

**DOI:** 10.7554/eLife.111684

**Published:** 2026-04-17

**Authors:** Shiyu Zhang, Min Zhou, Chi Shao, Yu Zhao, Mingzhe Liu, Lei Ni, Zhiyao Bao, Qiurui Zhang, Ting Zhang, Qun Luo, Jieming Qu, Zuojun Xu, Wei Zuo

**Keywords:** Human

Zhang S, Zhou M, Shao C, Zhao Y, Liu M, Ni L, Bao Z, Zhang Q, Zhang T, Luo Q, Qu J, Xu Z, Zuo W. 2025. Autologous P63+ lung progenitor cell transplantation in idiopathic pulmonary fibrosis: a phase 1 clinical trial. *eLife* **13**:RP102451. doi: 10.7554/eLife.102451.

Published 4 March 2025

[Details of correction]

The Results section and Table 2 have been corrected. The original manuscript contained two minor inaccuracies that arose during the final consolidation of clinical records. In the Results section (“Safety analysis of REGEND001”), the description of fever-related adverse events has been corrected from “1 grade II” to “2 grade II”. In Table 2 (Adverse events), the value for “Hemoptysis” at 1 M was incorrectly reported as 2 (16.67) and has been corrected to 1 (8.33). These corrections are limited to specific data points and do not affect the overall results, statistical analyses, or conclusions of the study.

[Results: Safety analysis of REGEND001]

[Corrected text:]

Fever is one of the mostly common cell therapy-related AEs (21.4%, 2 grade II, 2 grade I)

[Original text:]

Fever is one of the mostly common cell therapy-related AEs (21.4%, 1 grade II, 2 grade I)

[Table 2:]

[The corrected Table 2 is shown here:]

Table 2. Adverse events*

**Table:** 

	No. (%) of Patients With Adverse Events			
**Events**	**0.6** M	**1** M	**2** M	**3.3** M
Any adverse event	2 (16.67)	3 (25.00)	3 (25.00)	3 (25.00)
Serious adverse events	2 (16.67)	0	1 (8.33)	1 (8.33)
Fatal adverse events	0	0	0	0
Adverse events leading to discontinuation	0	0	0	0
Frequent adverse events:^†^				
Likely related to Bronchoscopy				
Hemoptysis	1 (8.33)	1 (8.33)	1 (8.33)	1 (8.33)
Fever	0	1 (8.33)	2 (16.67)	1 (8.33)
White blood cell counts increased	0	1 (8.33)	0	1 (8.33)
Productive cough	1 (8.33)	1 (8.33)	0	0
Bronchitis	1 (8.33)	0	0	0
Others				
COVID-19	0	0	3 (25.00)	3 (25.00)
Bronchitis	2 (16.67)	0	0	1 (8.33)
Hypokalemia	1 (8.33)	0	1 (8.33)	0

*Shown are adverse events occurred in patients from baseline examination to the end of the study visit. Events are listed in descending order of frequency.

†The frequent adverse events were defined as those with an incidence ≥2 patients, which were ordered by frequency of occurrence here.

[The originally published Table 2 is also shown for reference:]

Table 2. Adverse events*

**Table:** 

	No. (%) of Patients With Adverse Events			
**Events**	**0.6** M	**1** M	**2** M	**3.3** M
Any adverse event	2 (16.67)	3 (25.00)	3 (25.00)	3 (25.00)
Serious adverse events	2 (16.67)	0	1 (8.33)	1 (8.33)
Fatal adverse events	0	0	0	0
Adverse events leading to discontinuation	0	0	0	0
Frequent adverse events:^†^				
Likely related to Bronchoscopy				
Hemoptysis	1 (8.33)	2 (16.67)	1 (8.33)	1 (8.33)
Fever	0	1 (8.33)	2 (16.67)	1 (8.33)
White blood cell counts increased	0	1 (8.33)	0	1 (8.33)
Productive cough	1 (8.33)	1 (8.33)	0	0
Bronchitis	1 (8.33)	0	0	0
Others				
COVID-19	0	0	3 (25.00)	3 (25.00)
Bronchitis	2 (16.67)	0	0	1 (8.33)
Hypokalemia	1 (8.33)	0	1 (8.33)	0

*Shown are adverse events occurred in patients from baseline examination to the end of the study visit. Events are listed in descending order of frequency.

†The frequent adverse events were defined as those with an incidence ≥ 2 patients, which were ordered by frequency of occurrence here.

The article has been corrected accordingly.

