# Supplementary material for: Autologous P63+ lung progenitor cell transplantation in idiopathic pulmonary fibrosis: a phase 1 clinical trial
Source: eLife. 2025 Mar 4;13:RP102451. doi: 10.7554/eLife.102451 (PMC11879106; doi:10.7554/eLife.102451)
Supplement: Supplementary file 1. [file elife-102451-supp1.pdf]

**Registration Number**	CTR20210349	**Trial Status**	Completed
**Applicant**	Ting Zhang	**Initial Disclosure Date**	2021-03-01
**Sponsor Name**	Regend Therapeutics XLotus (Jiangxi) Co, Ltd.		

1. Title and Background Information

**Registration Number**	CTR20210349
**Related Registration Number**	NA
**Drug Name**	REGEND001 Autologous Therapy Product
**Drug Categories**	Biological products
**Clinical Trial Application Number**	Private
**Indications**	Early and mid-stage idiopathic pulmonary fibrosis (IPF)
**Official Title**	An Open-label Clinical Study to Explore the Safety, Tolerability and Preliminary Efficacy of REGEND001 Autologous Therapy Product for Treatment of Idiopathic Pulmonary Fibrosis (IPF).
**Common Title**	Exploratory Study of REGEND001 Autologous Therapy Product for the Treatment of Idiopathic Pulmonary Fibrosis (IPF)
**Protocol Number**	XHYX-IND-IPF-P1
**Latest Version Number**	V4.0
**Date of Latest Version**	2021-05-18
**Combination Therapy**	No

2.Sponsor Information

**Sponsor Name**	Regend Therapeutics XLotus (Jiangxi) Co, Ltd.		
**Applicant**	Ting Zhang	**Landline Number**	021-60646685
**Mobile Number**	18516646727	**Email Address**	winnie-tzhang@regend.cn
**Contact Address**	Building 8, Nanchang National Pharmaceutical	**Contact Postcode**	330096

	International Innovation Park Joint Research Institute, 269 Aixi Lake North Road, High-tech Development Zone, Nanchang, Jiangxi Province		

3. Clinical Trial Information

**(1). Trial Objective**:	
Primary Objective:<br>To evaluate the safety and tolerability of different doses of REGEND001 autologous therapy product for the treatment of idiopathic pulmonary fibrosis (IPF).<br>Secondary Objective:<br>To assess the efficacy of different doses of REGEND001 autologous therapy product for the treatment of IPF and recommend appropriate cell therapy doses for subsequent clinical studies.	
**(2). Trial design**	
**Trial Classification**	Safety and efficacy
**Trial Phase**	Exploratory Phase 1 clinical trial
**Design Type**	Single-arm trial
**Randomization**	Non-randomized
**Blinding**	Open-label
**Trial Scope**	Domestic trials
**(3). Subject Information**	
**Age**	50 to 75 years old (Including ages 50 and 75)
**Sexes Eligible for Study**	Male and Female
**Accept Healthy Volunteers**	No
**Inclusion Criteria:**	1). Male or female, aged between 50 to 75 (Including ages 50 and 75);
	2). Subjects diagnosed with IPF according to guidelines for the diagnosis of idiopathic pulmonary fibrosis 2018 edition;
	3). Subjects with 30%~79% of the predicted value in diffusing capacity for carbon monoxide (DLCO) and more than 50% of the predicted value in forced vital capacity (FVC) in pulmonary function tests 3 months before screening;
	4). Subjects with typical High-resolution computed tomography (HR-

	CT) imaging findings of idiopathic pulmonary fibrosis in the past 12 months;
	5). Subjects tolerant to bronchofiberscope;
	6). Subjects fully informed of the purpose, method and possible discomfort of the trial, agreeing to participate in the test, and voluntarily signing the informed consent;
	7). Subjects with good adherence, willingness to take medication and regular follow-up examinations as required by the protocol ；
	8). Subjects able to understand and cooperate with the completion of pulmonary function tests.
**Exclusion Criteria:**	1) Subjects who cannot tolerate cell therapy
	2) Pregnant or lactating women;
	3) Subjects with syphilis or any of human immunodeficiency virus (HIV), hepatitis B virus (HBV), hepatitis C virus (HCV) positive antibody; Of which stable HBV carriers after drug treatment (DNA titer ≤500 IU/mL or copy number <1000 copies/mL) and cured hepatitis C patients (HCV RNA is negative) can be enrolled；
	4) Subjects with malignant tumors or a history of malignant tumors;
	5) Subjects with taking drugs which caused lung fibroblast such as amiodarone in a long term before screening;
	6) Subjects with infections in lung or other site, including bacterial and viral infections, requiring intravenous treatment before cell transplantation;
	7) Subjects with a history of invasive or noninvasive mechanical ventilation within 4 weeks;
	8) Subjects with any of the following lung diseases: asthma, active tuberculosis, pulmonary embolism, pneumothorax, pulmonary hypertension, pneumoconiosis, etc.; lung cancer, bronchiolitis obliterans or other active lung disease; Pneumonia currently or within the last 4 weeks; Pneumonectomy Previously;
	9) Subjects needing oxygen therapy currently (oxygen therapy time> 15h/d);
	10) Subjects suffering from serious other systemic diseases, such as myocardial infarction, unstable angina, liver cirrhosis, acute glomerulonephritis, connective tissue disease, etc.;
	11) Subjects with following results: leukopenia (leukopenia < 4×10^9/L) or agranulocytosis (leukocyte < 1.5×10^9/L or neutrophils < 0.5×10^9/L) of any cause; Blood creatinine > 2.5 times the upper limit of normal; Alanine transaminase (ALT) and Aspartate transaminase (AST) > 2.5 times the upper limit of

	normal values in the laboratory tests.
	12) Subjects with a history of mental illness or suicide risk, epilepsy or other central nervous system disorders
	13) Subjects with severe arrhythmias (such as ventricular tachycardia, frequent supraventricular tachycardia, atrial fibrillation, atrial flutter, etc.) or atrioventricular block of degree II or above, shown by 12-lead Electrocardiogram (ECG);
	14). Subjects with a history of abusing alcohol and illicit drug;
	15). Subjects who are allergic to cattle products;
	16). Subjects who participated in other clinical trials in the past 3 months;
	17). Subjects with poor compliance and difficult to complete the investigation;
	18). Investigators, employees of research centers or family members of them (none of whom are suitable to participate in the trial to ensure the objectivity of the research);
	19). Subjects who had an acute exacerbation of IPF or hospitalized for other respiratory diseases 3 or more times in the past 1 year;
	20). Subjects who take nintedanib for medication within 1 month, or plan to continue taking nintedanib for medication;
	21). Subjects with other acquired or congenital immunodeficiency disorders, or with a history of organ transplantation or cell transplant therapy;
	22). Subjects whose expected survival may be less than one year judged by the investigator;
	23). Male participants of childbearing potential and female participants within childbearing age were reluctant to use effective contraception from the time of signing the informed consent to 6 months after cell therapy;
	24). Subjects assessed as inappropriate to participate in this clinical trial by investigator.

(4). Interventions

**Investigational Drug**	**Serial Number**	**Name**	**Administration**
	1).	English Generic Name：REGEND001 Autologous Therapy Product	Dosage Form：Intratracheal administration preparation<br>Specifications：14mL/bag<br>Administration：Intratracheal administration through fiberoptic bronchoscopy

			Administration Frequency：Single dose
**Control Drug**	**Serial Number**	**Name**	**Administration**
		NA	NA

(5). Outcome Measures

**Primary Outcome and Assessment Time**	**Serial Number**	**Indicators**	**Assessment Time**	**Type of indicators**
	1)	Incidence and severity of the cell therapy-related adverse events (AEs)	Within 24 weeks after treatment	Safety Indicator

**Secondary Outcomes and Assessment Time**	**Serial Number**	**Indicators**	**Assessment Time**	**Type of indicators**
	1)	Incidence of complication related to bronchoscopy；	Within 24 weeks after treatment	Safety Indicator
	2)	Change of tumor markers from baseline	12 and 24 weeks after treatment	Safety Indicator
	3)	Change of routine safety assessments (Blood routine, Urine routine, Blood biochemistry, 12-lead Electrocardiogram (ECG))	Within 24 weeks after treatment	Safety Indicator
	4)	Change of the percentage of predicted value for single-breath diffusing capacity for carbon monoxide (DLCO-sb) from baseline	4, 12 and 24 weeks after treatment	Efficacy Indicator
	5)	Change of forced vital capacity (FVC) from baseline	4, 12 and 24 weeks after treatment	Efficacy Indicator
	6)	Change of the ratio of diffusing capacity for carbon monoxide/ the alveolar volume	4, 12 and 24 weeks after treatment	Efficacy Indicator

		(DLCO/VA) from baseline		
	7)	Change of 6-minute-walk test (6MWT) from baseline	4, 12 and 24 weeks after treatment	Efficacy Indicator
	8)	Change of St. George's respiratory questionnaire (SGRQ) scale from baseline	4, 12 and 24 weeks after treatment	Efficacy Indicator
	9)	Change of imaging of lung by high resolution computed tomography (HR-CT)	24 weeks after treatment	Efficacy Indicator
	10)	Idiopathic pulmonary fibrosis (IPF) exacerbation events (Frequency and severity)	Within 24 weeks after treatment	Efficacy Indicator
**(6). Has Data Monitoring Committee （DMC）**	No			
**(7). Trial injury insurance for subjects**	Yes			

4. Investigator Information

**(1). Principal Investigator Information**			
**Name**	Zuojun Xu	**Degree**	Medical Doctor (MD)
**Professional Title**	Senior	**Phone Number**	010-69156114
**Email**	xuzj@hotmail.com	**Postal Address**	No. 1, Shuaifu Garden, Dongcheng District, Beijing, Beijing Municipality
**Postal Code**	100730	**Institution Name**	Peking Union Medical College Hospital

**(2). Institution Information**				
**Serial Number**	**Institution Name**	**Principal Investigator**	**Country or Region**	**Province (State) - City**

1)	Peking Union Medical College Hospital	Zuojun Xu	China	Beijing Municipality - Beijing City
2)	Ruijin Hospital, Shanghai Jiao Tong University School of Medicine l	Jieming Qu	China	Shanghai Municipality - Shanghai City
3)	The First Affiliated Hospital of Guangzhou Medical University	Qun Luo	China	Guangdong Province - Guangzhou City

5. Ethics Committee Information

Serial Number	Name	Review Conclusion	Approval Date/Review Date
1)	Ethics Committee for Clinical Trials of Drugs at Peking Union Medical College Hospital Chinese Academy of Medical Sciences	Agreed after modification	2021-01-28
2)	Rapid review approval from the Ethics Committee for Clinical Trials of Drugs at Peking Union Medical College Hospital Chinese Academy of Medical Sciences	Agree	2021-06-07

6. Trial Status

(1). Trial Status	
Completed	
**(2). Participant Enrollment**	
**Target Enrollment**	Domestic: 24 participants；
**Enrolled Participants**	Domestic: 12 participants；
**Actual Total Enrollment**	Domestic: 12 participants；
**(3). Participant Enrollment and Study Completion Date**	
**Date of First Subject Signing Informed Consent**	Domestic：2021-05-14；
**Date of First Subject Enrollment**	Domestic：2021-07-19；
**Study Completion Date**	Domestic：2023-06-09；
